# Workshop on cardiovascular extracellular matrix in health and disease in Baeza, Spain

**DOI:** 10.1186/s13069-014-0018-1

**Published:** 2015-02-02

**Authors:** Enrique Lara-Pezzi, Elke Dworatzek, Fernando Rodríguez-Pascual

**Affiliations:** Cardiovascular Development and Repair Department, Centro Nacional de Investigaciones Cardiovasculares (C.N.I.C.), Madrid, Spain; Institute of Gender in Medicine (GiM) and Center for Cardiovascular Research (CCR), Charité-Universitaetsmedizin Berlin, Berlin, Germany; Centro de Biología Molecular “Severo Ochoa”, Consejo Superior de Investigaciones Científicas (C.S.I.C.), Universidad Autónoma de Madrid (U.A.M.), Nicolás Cabrera 1, E28049 Madrid, Spain

**Keywords:** Extracellular matrix, Cardiovascular, Fibrosis, Fibroblast, Myocardial infarction, Aneurysm, Vascular wall

## Abstract

The Workshop on Cardiovascular Extracellular Matrix in Health and Disease, International University of Andalusia, Baeza, Spain, 6-8 October 2014 served to discuss the current knowledge on the mechanisms integral to extracellular matrix homeostasis that are fundamental to understanding the pathological basis of several cardiovascular diseases, including the development of cardiac fibrosis in response to cardiac hypertrophy and myocardial infarction, and the extracellular matrix alterations contributing to aortic stenosis or aneurysms.

## Introduction

**Figure 1:**
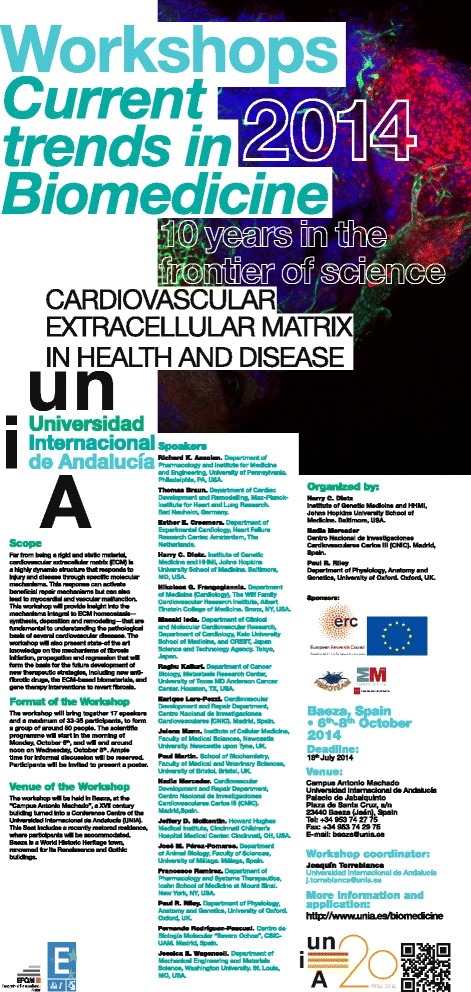
**Official poster announcement of the International University of Andalusia Workshop ‘Current Trends in Biomedicine’ on Cardiovascular Extracellular Matrix in Health and Disease, Baeza, Spain, 6-8 October 2014.** Image shows a cryoinjured zebrafish heart, image courtesy of Juan Manuel González-Rosa.

Clearly distinguishable from the glycocalix, the membrane-bound, carbohydrate-rich cell coat, the extracellular matrix (ECM) constitutes the organic matter found between most cells in plants and animals. Over recent years, our understanding of the functions of the ECM has evolved from the traditional concept of static ‘glue’, holding cells into tissues, to a more sophisticated view of a dynamic biomaterial that provides strength and elasticity, as well as anchor points of interactions with cell surface receptors, and availability of growth factors. Proper formation and organization of the ECM is essential for cell and tissue homeostasis. Matrix-related diseases arise from defects in the properties of ECM components, as in congenital diseases such as Marfan and Ehlers-Danlos syndromes, and from an excess of ECM production and deposition, generally termed as fibrosis. The International University of Andalusia Workshop ‘Current Trends in Biomedicine’ on Cardiovascular Extracellular Matrix in Health and Disease, was organized by Harry C. Dietz, Nadia Mercader and Paul R. Riley in Baeza, Spain, from 6 to 8 October 2014 with almost 50 attendees including basic as well as clinical scientists (Figure 1). The meeting aimed to bring together top-level, highly experienced investigators working on different aspects of cardiovascular ECM pathophysiology and mechanisms of fibrosis initiation, propagation, and regression in an attempt to shed some light on the yet challenging questions in the field and to progress into the development of new therapeutic strategies.

Four main sessions covered the role of fibrosis in cardiac remodeling, general mechanisms for tissue fibrosis, and the contribution of matrix remodeling to aortic diseases. A final session discussed novel therapeutics in cardiovascular diseases and tissue remodeling. The following sections will give an overview of the scientific content of the meeting.

### The cardiovascular extracellular matrix

In vertebrates, where the circulatory system consists of a central pump (the heart) and a network of tubes (the vasculature), through which the blood is continuously circulated, the ECM plays an essential role in determining the mechanical properties of the vascular and cardiac tissues. The main components of the cardiovascular ECM responsible for its biomechanical behavior include elastin and collagen. Considering the vascular scenarios where the blood pressure is at highest, namely the heart and the aorta, an exquisite combination of collagen and elastin provides the aortic tissue with its properties of strength and recoil, respectively. In the heart, cardiomyocytes are embedded in a collagen network, which is essential for contraction but is also implicated in the tissue response to damage.

### Fibrosis in cardiac remodeling

Extensive ECM remodeling occurs in the diseased heart. Irrespective of whether cardiomyocyte loss occurs because of an ischemic event, such as a myocardial infarction, or as a response to pressure or volume overload or the result of congenital cardiomyopathies, damage to the myocardium results in severe alterations of the cardiac collagen network. The molecular mechanisms governing these processes and the therapeutic opportunities that arise from their understanding are an active area of research.

Myofibroblasts constitute the main cellular effectors of cardiac fibrosis, and the activation of transforming growth factor-β (TGF-β) is likely the most important signaling pathway contributing to myofibroblast differentiation. Nikolaos G. Frangogiannis (Albert Einstein College of Medicine, USA) described the effects of suppressing Smad3 specifically in periostin-positive myofibroblasts. Periostin is not expressed in healthy adult hearts, but is markedly and selectively upregulated in activated injury-site myofibroblasts. The group of Frangogiannis observed accentuated cardiac remodeling associated with defective scar contraction in myofibroblast-specific Smad3 knockout mice. Interestingly, Smad3 loss perturbs spatial alignment of infarct myofibroblasts, a typical response linked to the deposition of long and highly aligned collagen fibers in the infarct area. An important issue, still a matter of intense debate, is the origin of myofibroblasts in the damaged heart. Onur Kanisicak from the group of Jeffery D. Molkentin (Cincinnati Children’s Hospital Medical Center, USA) approached the study of myofibroblast ontogeny by tracing fibroblasts in TCF21- and periostin-Cre mouse lines. According to their results, TCF21 labels quiescent fibroblasts within the adult heart. Upon myocardial damage, these cells first give rise to proto-myofibroblasts expressing both TCF21 and periostin, and finally to periostin-positive myofibroblasts committed to cardiac fibrosis and remodeling. Epicardial- and bone marrow-derived cells have also been shown to contribute to activated myofibroblasts within the damaged heart. José María Pérez-Pomares (University of Málaga, Spain) and Adrián Ruiz-Villalba (Academical Medical Center, The Netherlands) described the exquisite cell-cell interaction between both cell types for patterned collagen deposition, being epicardial-derived fibroblasts mostly responsible for collagen expression. Inflammatory cells such as macrophages are also important players in the tissue response to heart damage. Thomas Braun (Max-Planck-Institute for Heart and Lung Research, Germany) showed the potential of the macrophage cytokine oncostatin M to dedifferentiate cardiomyocytes and thereby providing protective effects during myocardial infarction.

In contrast to adult mammals, an impressive regenerative capacity has been demonstrated in the hearts of zebrafish and newts. These animals display a robust proliferative competence of cardiomyocytes in adult life, which provides a source to form a new myocardium after injury. Nadia Mercader (Centro Nacional de Investigaciones Cardiovasculares, Spain) described the potential of models of cryoinjury-induced heart damage in zebrafish to elucidate the processes underlying the regeneration capacity, with particular interest in the origin of cells contributing to scar formation and regression. Rebeca Richardson from the group of Paul Martin (University of Bristol, United Kingdom) emphasized also the remarkable competence of embryos and adult teleosts for complete, scar-less tissue regeneration, not only in heart but also in skin, and the important role of the inflammatory cell response to complete this process.

Research on specific ECM or ECM-associated proteins is revealing also important clues on tissue remodeling during heart damage. Nathalie Pizzinat (Institute of Cardiovascular and Metabolic Diseases, France) discussed the role of the ECM glycoprotein tenascin C in pressure overload-induced cardiac dysfunction. Tenascin C deficient mice show reduced accumulation of inflammatory cytokines and chemokines as well as diminished leukocyte recruitment upon heart damage, compared to wild type animals. Additionally, Fernando Rodriguez-Pascual (Centro de Biología Molecular, Spain) discussed the contribution of matrix crosslinking lysyl oxidases to collagen scar formation and cardiac dysfunction upon myocardial remodeling. As shown by his group, inhibition of lysyl oxidase enzymatic activity impaired collagen cross-linking, an effect associated with improved cardiac function.

An interesting presentation from Elke Dworatzek (Universitaetsmedizin Berlin, Germany) showed dimorphic collagen expression in male *versus* female pressure overload-induced hearts, an observation probably relying on differential estrogen-mediated signaling in cardiac fibroblasts.

Finally, the development of cardiac fibrosis is also regulated by the actions of miRNAs. This was highlighted by Esther Creemers (Academic Medical Center, The Netherlands), who described the capacity of miRNA-15 family to regulate TGF-β signaling and thereby to control cardiac fibrosis.

### Fibrosis in other tissues helps to decipher cardiac fibrosis

While undoubtedly showing tissue-specific features, cardiac fibrosis is also governed by general mechanisms of tissue fibrosis. An example of a molecular pathway contributing to fibrosis in a global way is the transient receptor potential canonical member 6 (TRPC6), an integral membrane calcium channel described to be essential for cardiac, dermal, and embryonic fibroblasts to transdifferentiate into fibrosis-competent myofibroblasts, as shown by Molkentin. Pura Muñoz-Canoves (Pompeu Fabra University and ICREA, Spain) has studied the issue of the myofibroblast origin in the context of muscular dystrophies. Distinct genetically engineered mice enabled her group to trace and map the origin of these cells. They proposed that, in addition to resident fibroblasts, myoblast cells and other resident and infiltrating cell types within skeletal muscle transit into myofibroblasts during muscular dystrophy. To this respect, Raghu Kalluri (University of Texas, USA) revised the concept of cellular plasticity in the pathogenesis of fibrosis, with particular emphasis on the contribution of the endothelial-to-mesenchymal transition to this process. Jelena Mann (University of Newcastle, United Kingdom) discussed the influence of epigenetics to regulate the progression of chronic liver fibrosis. She showed how an epigenetically regulated fibrotic signature can be transmitted from one generation to its offspring, and from one organ to another within an organism.

### Matrix remodeling in aortic diseases

In the arterial wall, elastin and collagen deposited mainly (but not exclusively) by vascular smooth muscle cells provide the tissue with specific biomechanical features. Elastin organizes into a 3D-interconnecting lamellar network designed to transfer the tremendous surge of pressure as blood is ejected from the heart throughout the vessel wall. On the other hand, bundles of collagen between the lamellar layers progressively restrict aortic distension at high pressures, contributing to the non-linear nature of vascular elasticity, which is essential for preventing distension and further damage to the vessel with increased pressure. Alterations in ECM homeostasis have been invoked to be the causative factor of a number of aortopathies, including the formation of aortic aneurysms or the development of aortic stiffness.

A paradigm disease in aortic pathology is Marfan syndrome (MFS), a hereditary connective tissue disorder caused by mutations in the fibrillin-1 gene. Harry C. Dietz (Johns Hopkins University, USA) discussed the fundamental role of the overactivation of TGF-β signaling in the initiation and progression of the aneurysmal disease in MFS, and the potential of the angiotensin receptor blocker, losartan to ameliorate the aortic pathology. In the search for gene modifiers, he introduced MAS1, an antagonist of angiotensin signaling, and mitogen-activated protein kinase kinase kinase 4 (MAP3K4), a central regulator of MAPK pathway, as potential modifying loci for MFS severity. Several investigations including those from the group of Dietz situate TGF-β signaling downstream of angiotensin activation along a pathway leading to aortic dilatation in MFS. Nevertheless, refinements of the mouse models by Jason R. Cook from the group of Francesco Ramirez (Icahn School of Medicine at Mount Sinai, USA) showed that both pathways could differentially contribute to the initiation and progression of the aortic dilatation, with mechanical stress playing a fundamental role in the activation of angiotensin type 1 receptor in a ligand independent manner. Additionally, Gustavo Egea (University of Barcelona, Spain) described the TGF-β-dependent phenotypic modulation of vascular smooth muscle cells in MFS patients and mice. Furthermore, Rodriguez-Pascual showed experimental results indicating a protective role for matrix crosslinking lysyl oxidases in the formation of aortic dilatation in MFS mouse models.

MFS and related disorders including Loeys-Dietz or aneurysm-osteoarthritis syndromes account for less than 5% of known thoracic aortic aneurysms. Genetic abnormalities explain about 20% of the remaining 95% of patients, and several studies have identified mutations in proteins within the ECM and the contractile machinery associated with thoracic aortic aneurysms. However, many gene mutations remain to be discovered. Juan Miguel Redondo (Centro Nacional de Investigaciones Cardiovasculares, Spain) showed experimental results indicating an important role for the peptidase of the ADAMTS (a disintegrin and metalloproteinase with thrombospondin motif) family, ADAMTS1 in the molecular events contributing to the formation of aortic aneurysms, suggesting that mutations within this gene may be involved in human aortic pathology.

Mechanical forces dominate the performance of the vessel wall, especially in the large, elastic arteries. Jessica Wagenseil (Washington University, USA) emphasized the importance of this concept by showing experiments in elastin- and fibulin-4 null mice. According to her results, the development of aortic stenosis or aneurysms in these mouse models may be determined by smooth muscle cells from distinct embryonic origins that respond differently to mechanical forces.

Finally, Rick Assoain (University of Pennsylvania, USA) described the importance of the matrix metalloproteinase (MMP) 12 in the development of aortic stiffness in cardiovascular diseases and during aging.

### Novel therapeutics in cardiovascular diseases and tissue remodeling

The ultimate aim of the investigation on ECM contribution to cardiovascular diseases is the development of novel therapeutic strategies. Different promising approaches have been presented during the workshop. Enrique Lara-Pezzi (Centro Nacional de Investigaciones Cardiovasculares, Spain) explained the capacity of the naturally occurring splicing variant of the calcineurin catalytic subunit (CnAβ1), to ameliorate cardiac dysfunction in mouse models of myocardial infarction and pressure overload-induced hypertrophy, indicating CnAβ1 as an interesting candidate for gene therapy in the heart. A systematic approach to identify small molecules displaying regenerative capacity in *in vitro* models of heart damage was presented by Paul R. Riley (University of Oxford, United Kingdom). Finally, Masaki Ieda (Keio University School of Medicine, Japan) revised the iterative process that led to the identification of the transcription factor cocktail reprogramming fibroblasts into cardiomyocytes as an attempt to develop therapeutic strategies for heart repair and regeneration.

## Concluding remarks

A highly interactive program of 3 days of duration, which included full communications, short talks and poster presentations, encouraged an interesting exchange of ideas among scientists working on different diseases and experimental models related to cardiovascular ECM pathophysiology. The meeting unveiled the intense efforts that are being carried out to decipher the molecular players and cell types responsible for the development of cardiac and vascular fibrosis. Improving our understanding of these mechanisms will undoubtedly contribute to the development of new therapeutic strategies to tackle fibrosis in cardiovascular diseases and other related conditions.

## Abbreviations

ADAMTS: A disintegrin and metalloproteinase with thrombospondin motif
CnAβ1: calcineurin A subunit β1
ECM: Extracellular matrix
MAP3K4: Mitogen-activated protein kinase kinase kinase 4
MFS: Marfan syndrome
TGF-β: Transforming growth factor-β
TRPC6: Transient receptor potential canonical member 6

